# Venous blood gases and electrolyte values of captive red foot tortoise (*Chelonoidis carbonarius*)

**DOI:** 10.1371/journal.pone.0299451

**Published:** 2024-03-15

**Authors:** Sofia Silva La Rocca de Freitas, Laís Velloso Garcia, Jairo Antonio Melo dos Santos, Líria Queiroz Luz Hirano

**Affiliations:** 1 Resident of Wild Animals Medicine at Federal University of Uberlândia (UFU), Uberlândia, Minas Gerais, Brasil; 2 Veterinary at University at Brasilia (UnB), Brasília, Brasil; 3 Chief Executive Officer at S.E.V.VO, Brasília, Brasil; 4 Professor of the Graduate Program in Animals Sciences at University of Brasilia (UnB), Brasília, Brasil; University of Minnesota, UNITED STATES

## Abstract

Blood gas analysis reflects the exchange of oxygen and carbon dioxide in the lungs. This test provides important information, since the relationship between these gases has a direct impact on the acid-basic balance in the body. Given the significance of blood gas analysis in Brazilian reptiles, this study set out to establish temperature-corrected and uncorrected reference intervals for venous blood gas measurements in *Chelonoidis carbonarius*, and to compare values between females and males. In this study, 19 animals were used, 8 males and 11 females. Blood samples were collected from the dorsal coccygeal vein, and the analyses were performed immediately after blood sample collection. The following parameters were measured: pH, PO_2_, HCO_3_^-^, TCO_2_, BEecf, Na, K, ICa, and Glu, and were compared between females and males. Additionally, pH, pCO_2_, and pO_2_ values were compared with and without temperature correction. Oxygen saturation and Na levels were significantly higher (p<0.05) in males. Furthermore, it was possible to infer that the lower the body temperature relative to the environmental temperature, the larger the difference in pH following temperature correction.

## Introduction

Blood gas analysis reflects the exchange of oxygen and carbon dioxide in the lungs. This test provides important information, since the relationship between these gases has a direct impact on the acid-basic balance in the body. Abnormal blood gas levels may indicate respiratory, metabolic or renal disorders. Venous blood gas values are similar to arterial blood values. The only exception is partial pressure of oxygen, which is significantly lower in venous blood due to oxygen consumption in metabolic processes [1].

Body temperature affects the concentration of most blood gases. Ceretta et al. [2] compared venous blood gas measurements with and without temperature correction in *Agkistrodon contortrix* and *Pantherophis alleghaniensis*. Temperature correction yielded higher pH values, whereas remaining parameters tended to be lower when temperature correction was used.

According to Lewbart et al. [3], the establishment of species-specific parameters is particularly important in reptiles due to adaptive variations driven by environmental factors. Blood gas values have been reported in *Agkistrodon contortrix*, *Pantherophis alleghaniensis* [2], *Chelonia mydas* [4], *Caretta caretta* [5] and *Amblyrhynchus cristatus* [3]. However, despite the growing relevance of blood gas analysis in reptile medicine, there is a limited amount of data, especially on native Brazilian testudines.

In Brazil, there are two native tortoise species, *Chelonoidis carbonarius* Spix, 1824 and *C*. *denticulatus*. These omnivore reptiles found in warm climates have thick skin and scales to prevent dehydration, and are able to absorb water through the cloaca before excretion. In these oviparous animals, sexual dimorphism is evident in sexually mature individuals: males have a concave dip in their plastron, whereas the female plastron is flat [6].

Given the significance of blood gas analysis in Brazilian reptiles, this study set out to establish emperature-corrected and uncorrected reference intervals for venous blood gas measurements in *Chelonoidis carbonarius*, and to compare values between females and males.

## Methods

This study was submitted to SISBIO (System of Approval and Biodiversity Information) and approved by the Ethics Committee of University of Brasilia (UnB). Nineteen healthy adult *Chelonoidis carbonarius* specimens (8 males and 11 females) obtained from the Wildlife Triage Center of the Federal District (CETAS-DF) were used. Animals were rescued between 2019 and 2020.

Animals were submitted to clinical evaluation for determination of hydration status, level of activity and consciousness, and body condition score. All specimens were considered healthy based on clinical evaluation findings. Plastron size measurements were made using a measuring tape. Mean plastron length was 20.95 ± 5.85 cm in females and 28.13 ± 3.44 cm in males. Although their exact age could not be determined, all specimens had a similar size and were considered sexually mature for the species, *as per* Barros et al. [7].

Tortoises were numbered 1 to 19 using adhesive tape. Cloacal temperature (CT) was measured using a digital thermometer (B-Max Tp101, temperature range between -50°C and 300°C, precision 0.1°C, accuracy ±1%, Shenzhen, GD, China) with the probe inserted 50 mm into the cloaca. Venous blood samples were collected between 11 a.m. and 4 p.m. at a mean environmental temperature of 26 ± 3.55°C. Environmental temperature was controlled using a digital thermohygrometer (Incoterm 1005, temperature range between -50°C and 70°C, precision 0.1°C, accuracy ±1%, Porto Alegre, RS, Brazil).

Animals were restrained in the supine position and blood samples (0.5 mL) collected from the dorsal coccygeal vein into 1 mL heparinized syringes (sodium heparin 5,000 IU/L; Eurofarma, Ribeirão Preto—SP, Brazil). Blood gas analyses were performed immediately after blood sample collection using a blood gas analyzer with automatic calibration and temperature control (Abbot VETSCAN i-*STAT;* Abaxis Europe, Griesheim, Germany).

The following parameters were measured: partial pressure of carbon dioxide (pCO_2_), potential of hydrogen (pH), partial pressure of oxygen (pO_2_), base deficit (BEecf), bicarbonate (HCO_3_^-^), total carbon dioxide (TCO_2_), oxygen saturation (sO_2_), sodium (Na), potassium (K), ionised calcium (iCa) and glucose (Glu). Temperature-corrected and uncorrected, pH, pCO_2_ and pO_2_ values were estimated. Since tortoises did not receive supplemental oxygen, the fraction of inspired oxygen (FiO_2_) was set at 21% (atmospheric oxygen concentration) [8].

Data were entered into Excel spreadsheets (Microsoft Excel 16) and submitted to statistical analysis using BioEstat 5.3 [9]. Normal data distribution was confirmed using the Shapiro-Wilk test. Extreme values were determined based on deviations and outliers excluded. Analysis of variance (ANOVA) was used to compare means between males and females. The paired t-test was used to compare mean temperature-corrected and uncorrected pH, pCO_2_ and pO_2_ values. The level of significance was set at 5%.

## Results

Blood gas values obtained in this study are shown in Table 1. Oxygen saturation and Na levels were significantly higher (p<0.05) in males. Remaining parameters did not differ significantly between males and females.

**Table 1:** Venous blood gas analysis data for male and female specimens of *Chelonoidis carbonarius*.

	**MALES**							
** **		**MEAN**	**MEDIAN**	**STANDARD DEVIATION**	**STANDARD ERROR**	**MINIMUM**	**MAXIMUM**	**p M x F** ^*^
**pH**		7.43	7.41	4.54	1.6	7.39	7.48	0.2335
**pH+TC** (mmHg)		7.54	7.56.	1.61	5.69	7.27	7.76	0.213
**PCO**_**2**_ (mmHg)		33.39	33.60	12.41	4.39	11.1	55.9	0.1069
**PCO**_**2**_**+TC** (mmHg)		21.96	22.1	9.73	3.44	7.2	38.5	0.1072
**PO**_**2**_ (mmHg)		66.25	66.50	12.01	4.24	45	80	0.6755
**PO**_**2**_**+TC** (mmHg)		33.14	36	10.49	3.71	13	46	0.6021
**BEecf** (mmol/L)		-4.25	-2	7.13	2.52	-18	4	0.9947
**HCO**_**3**_^**-**^ (mmol/L)		22.47	23.1	3.38	1.2	17.1	27.3	0.1388
**TCO**_**2**_ (mmol/L)		23.57	24	3.26	1.15	18	28	0.1135
**sO**_**2**_(%)		91.87	93.5	4.76	16.80	84	96	< 0.0001
**Na** (mmol/L)		126.71	127	1.7	0.60	124	129	0.0249
**K** (mmol/L)		3.34	3	0.81	0.29	2.4	4.5	0.3253
**iCa** (mmol/L)		1.17	0.95	0.73	0.26	0.33	2.44	0.8428
**Glu** (mg/dL)		45.57	48	7.57	2.67	34	56	0.6931
**CT** (°C)		26.24	26.7	3.55	1.25	19.2	30.5	0.6825
	**FEMALES**							
		**MEAN**	**MEDIAN**	**STANDARD DEVIATION**	**STANDARD ERROR**	**MINIMUM**	**MAXIMUM**	
**pH**		7.51	7.47	1.94	0.58	7.26	7.86	
**pH+TC**		7.69	7.59	1.96	0.59	7.52	8.03	
**PCO**_**2**_ (mmHg)		24.45	23.3	10.46	3.15	11.1	39.2	
**PCO**_**2**_**-+TC** (mmHg)		15.54	16.10	6.33	1.91	7.3	25.2	
**PO**_**2**_ (mmHg)		70.3	64.5	26.73	8.05	35	113	
**PO**_**2**_**+TC** (mmHg)		37.30	29.00	18.57	5.59	15	67	
**BEecf** (mmol/L)		-4.27	-4	7.34	2.21	-14	10	
**HCO**_**3**_^**-**^ (mmol/L)		18.79	19.1	5.6	1.69	9.3	27.6	
**TCO**_**2**_ (mmol/L)		19.64	20	5.61	1.69	10	28	
**sO**_**2**_ (%)		90.3	93.5	10.15	3.05	68	100	
**Na** (mmol/L)		123.43	123	2.94	0.88	120	128	
**K** (mmol/L)		2.98	2.9	0.62	0.19	2.3	3.7	
**iCa** (mmol/L)		1.1	0.86	0.71	0.21	0.25	2.24	
**Glu** (mg/dL)		53.6	48	21.77	6.56	30	92	
**CT** (°C)		26.81	27.4	2.46	0.74	22.3	30.8	

*p M x F = p value in the mean comparison between males and females according to the paired t test, with a significance of 5%.

+TC = with temperature correction; BEecf = Base excess in extracellular fluid; Glu = Glucose; HCO_3_^-^ = Bicarbonate; iCa = Calcium ion; K = Potassium; Na = Sodium; PCO_2_ = Partial pressure of carbon dioxide; pH = Hydrogen potential; PO_2_ = Partial pressure of oxygen; sO_2_ = Oxygen saturation; CT = Cloacal temperature; TCO_2_ = Total carbon dioxide.

Comparative analysis of pH, pO_2_, and pCO_2_ values revealed significant differences in pO_2_ in males, with lower values obtained following temperature correction. In contrast, all three parameters differed significantly (p < 0.0001) in females. Temperature-correction tended to yield higher pH and lower pO_2_ and pCO_2_ values, regardless of gender (Table 2).

**Table 2:** Temperature-corrected (+TC) and uncorrected (-TC) pH, pO_2_, and pCO_2_ values obtained in male and female specimens of *Chelonoidis carbonarius* submitted to venous blood gas analysis.

	MALES			FEMALES		
	MEAN (-TC)	MEAN (+TC)	p*	MEAN (-TC)	MEAN (+TC)	p*
**pH** (mmHg)	7.43	7.54	0.0814	7.51	7.69	< 0.0001
**PCO**_**2**_ (mmHg)	33.39	21.96	0.1198	24.45	15.54	< 0.0001
**PO**_**2**_ (mmHg)	66.25	33.14	< 0.0001	70.3	37.30	< 0.0001

*p = p value in the mean comparison between males and females according to the paired t test, with a significance of 5%.

-TC = without temperature correction; +TC = with temperature correction.

## Discussion

Studies addressing blood gas analysis in reptiles have been published by Harms et al. [5], Lewbart et al. [4], Lewbart et al. [3] and Ceretta et al. [2]. In those studies, blood samples were obtained from the subcarapacial venous sinus, jugular vein, coccygeal arch and ventral coccygeal vein respectively. No publications describing arterial blood gas analysis in reptiles were found, probably due to technical difficulties associated with arterial puncture in these animals.

Temperature-corrected pH and pCO_2_ values measured in *C*. *carbonarius* in this study differed from values reported in *A*. *contortrix* (pH 0.18 and pCO_2_ 1.2 mmHg) and *P*. *alleghaniensis* (pH 0.13 and pCO_2_ 1.9 mmHg) [2]. In *Caretta caretta*, pH values of 0.13 and pCO_2_ values of 28.7 have been described [5]. In this sample, pH values of 0.18 and 0.115 and pCO_2_ values of 8.91 mmHg and 11.43 mmHg were obtained in females and males respectively. Higher temperature-corrected pH values have been reported in studies with other reptile species. In those studies, the lower the body temperature relative to the ambient temperature, the larger the difference in pH following temperature correction [2–5].

Studies comparing blood gas measurements in male and female reptiles have not been published to date. In this study, the values of sO_2_ were significantly higher in males than in females. This finding may be explained by higher red blood cell counts and hematocrit in males in response to androgens. Erythropoiesis is inhibited by estrogens and stimulated by testosterone. Hence the higher number of red blood cells available for oxygen transport in the blood in males [10].

According to O’Malley [11], testudines are the vertebrates with the highest levels of bicarbonate in the blood, which helps to buffer lactic acid produced during anaerobic respiration. Findings of this study support that hypothesis. Bicarbonate levels of 8.1 and 16.9 mmol/L have been reported in *A*. *contortrix* and *P*. *alleghaniensis* respectively, compared to 22.47 mmol/L in male and 18.79 mmol/L in female *C*. *carbonarius* specimens in this sample [2]. Even higher blood bicarbonate levels (43.8 mmol/L) have been described in *C*. *mydas*, a marine turtle species which can survive prolonged apnea, suggesting aquatic testudines are able to neutralize higher amounts of lactic acid. Shorter apneic periods in *C*. *carbonarius* are consistent with the terrestrial habits of this species [4].

Ionised Ca, sodium, and pH values in *C*. *carbonarius* specimens in this study did not differ from *A*. *contortrix* or *P*. *alleghaniensis* [2]. However, lower iCa values have been reported in *C*. *mydas* [4]. Potassium values tend to be lower in tortoises than in snake species [2]. Differences in potassium levels may reflect the physiology of feeding and excretion in terrestrial reptile species. Reptiles excrete uric acid, an evolutionary adaptation to prevent water loss and dehydration, and urate salts are composed of potassium in herbivorous species [12]. Although tortoises are omnivorous, they have a predilection for vegetarian diets (i.e., tend to be herbivores). Since urate salts consist primarily of potassium, serum potassium levels may be lower in tortoises when kidney function is normal [12, 13].

## Conclusions

This study provides reference intervals for venous blood gas analysis in *Chelonoidis carbonarius* and reveals significant gender-related differences in oxygen saturation and sodium levels, with higher values in males. It was also possible to infer that the lower the body temperature relative to the environmental temperature, the larger the difference in pH following temperature correction.
